# Colorimetric Detection of Organophosphate Pesticides Based on Acetylcholinesterase and Cysteamine Capped Gold Nanoparticles as Nanozyme

**DOI:** 10.3390/s21238050

**Published:** 2021-12-02

**Authors:** Muhammad Musaddiq Shah, Wen Ren, Joseph Irudayaraj, Abdulrahim A. Sajini, Muhammad Ishtiaq Ali, Bashir Ahmad

**Affiliations:** 1 Department of Biological Sciences, International Islamic University, Islamabad 44000, Pakistan; musaddiq.phdbt74@iiu.edu.pk; 2 Department of Bioengineering, The University of Illinois at Urbana-Champaign, Urbana, IL 61801, USA; wenren@illinois.edu (W.R.); jirudaya@illinois.edu (J.I.); 3 Biomedical Research Center, Mills Breast Cancer Institute, Carle Foundation Hospital, Urbana, IL 61801, USA; 4 Micro and Nanotechnology Laboratory, The University of Illinois at Urbana-Champaign, Urbana, IL 61801, USA; 5 Department of Biomedical Engineering, The Khalifa University, Abu Dhabi 127788, United Arab Emirates; abdulrahim.sajini@ku.ac.ae; 6 Department of Microbiology, Quaid-i-Azam University, Islamabad 45320, Pakistan; ishimrl@qau.edu.pk

**Keywords:** acetylcholinesterase inhibitors, pesticide intoxication, colorimetric biosensor, neurotoxin, nanozyme, parathion ethyl

## Abstract

Organophosphates (OPs) are neurotoxic agents also used as pesticides that can permanently block the active site of the acetylcholinesterase (AChE). A robust and sensitive detection system of OPs utilising the enzyme mimic potential of the cysteamine capped gold nanoparticles (C-AuNPs) was developed. The detection assay was performed by stepwise addition of AChE, parathion ethyl (PE)-a candidate OP, acetylcholine chloride (ACh), C-AuNPs, and 3, 3′, 5, 5′-tetramethylbenzidine (TMB) in the buffer solution. The whole sensing protocol completes in 30–40 min, including both incubations. The Transmission Electron Microscopy (TEM) results indicated that the NPs are spherical and have an average size of 13.24 nm. The monomers of C-AuNPs exhibited intense catalytic activity (nanozyme) for the oxidization of TMB, revealed by the production of instant blue colour and confirmed by a sharp peak at 652 nm. The proposed biosensor’s detection limit and linear ranges were 5.8 ng·mL^−1^ and 11.6–92.8 ng·mL^−1^, respectively, for PE. The results strongly advocate that the suggested facile colorimetric biosensor may provide an excellent platform for on-site monitoring of OPs.

## 1. Introduction

Organophosphates (OPs) are commonly used as chemical warfare agents and pesticides in developing countries due to their low cost, wide availability, and high potency [1,2]. Due to their inclusive efficacy as insecticides (methyl parathion, chlorpyrifos), herbicides (glyphosate, diuron), nematicide (carbofuran, carbaryl), and fungicides (imazalil, thiabendazole); the OP pesticides have extensively been applied in agricultural pest control. The residues of the OPs, which have a long persistence in the natural environment, pose a severe threat to terrestrial and aquatic animals [3]. Even low-dose exposures of OPs for a long time can induce adversative health effects on fauna [4]. According to the U.S. Environmental Protection Agency, OPs are highly toxic and categorized as the most toxic “Class 1” compound [5,6]. The presence of OPs leads to the malfunctioning of the central and peripheral nervous system by blocking the active site of AChE (EC 3.1.1.7), a prime functioning enzyme of the nervous system and the neurotransmitters. The enzyme controls the level of acetylcholine (ACh) by catalysing it into choline and acetate at the synapse, which leads to the culmination of neurotransmission. Accumulation of ACh interferes with muscular functions, respiration, fluctuation in blood pressure, myocardial abnormalities, seizures, and ultimately death [7,8].

A number of approaches have been adopted to detect the OPs as mentioned in the Table 1. Detection of OP compounds has been traditionally performed with analytical methods such as gas chromatography (GC) [9], high-performance liquid chromatography (HPLC) [10], capillary electrophoresis [11], surface plasmon resonance [12], fluorimetry [13], and spectroscopic methods [14,15]. Although these techniques have been standardized, none of these methods is suitable for on-site monitoring and rapid detection of OPs [16]. Biosensor-based detection of neurotoxins has the potential to be easy, rapid, cost-effective, portable, specific, and highly sensitive without the need for lengthy procedures [7]. Electrochemical biosensors for OPs detection have proven successful biosensing methods because of simple instruments, high reliability, and compatibility with complex samples [17,18].

Among biosensing detection methods, enzyme-based biosensors can be an excellent alternative for detecting insecticides and pesticides, including OPs, because these toxins are enriched with compounds that can block the diverse enzymes of insects and pests [19], including AChE. Oxidase mimicking materials such as MnO_2_ nanosheets and surface-modified cerium oxide nanoparticles have been used to generate signals for detection of OPs by colorimetry via TMB oxidation [20,21]. TMB, nanozymes, and hydrogen peroxide (H_2_O_2_) based colorimetric detection systems have been employed in prior works [22,23,24].

The term “nanozyme” was coined first time to describe gold nanoparticles (AuNPs) possessing the capability of transphosphorylation [25]. Nanozymes are those nanomaterials capable of mimicking the functions of enzymes [26] as these easy-to-synthesise materials exhibited higher stability than proteins under harsh conditions [27,28]. Consequently, their applications in environmental monitoring [29,30] are highly desirable. Enzyme mimicking materials can be categorized into two classes, oxidoreductases and hydrolases [31]. Colorimetric nanozyme based biosensing of OPs is a simple approach due to the on-site monitoring of these compounds with naked eyes, and without the requirements of readout instrument.

In the current study, the peroxidase-like activity of C-AuNPs was exploited to detect PE by a convenient colorimetric assay. C-AuNPs were able to produce some definite colorimetric response with a chromogenic agent (TMB). Additionally, the C-AuNPs provided indirect indication about functional and non-functional states of AChE. The distinctive feature of the developed method is that naked-eye detection of PE is plausible by monitoring the colour of the reaction.

## 2. Materials and Methods

AChE (from *Electrophorus electricus),* ACh, gold salt (HAuCl_4_·3H_2_O)_,_ and PE were purchased from Sigma Aldrich, St. Louis, MO, USA. TMB was obtained from MOSS Inc., Chicago, IL, USA. All chemicals were of analytical grade and used as received.

### 2.1. Synthesis of C-AuNPs

Standard and working solutions of HAuCl_4_·3H_2_O, cysteamine, and sodium borohydride (NaBH_4_) were prepared using Milli Q water (18.25 MΩ·cm) in volumetric flasks. C-AuNPs were synthesized by adopting the method, with necessary modifications elaborated by Ren et al. [42]. Briefly, 1.42 mM solution (40 mL) of HAuCl_4_·3H_2_O was sonicated for 5.0 min and mixed with 400 µL of cysteamine solution at a concentration of 213 mM, followed by continuous stirring at 500 rpm for 20 min in an amber flask. A capping solution consisting of 10 mM (10 µL) NaBH_4_ was added and kept at constant stirring for an additional 30 min at room temperature. To remove the unreacted species and unbound cysteamine ligands from colloidal C-AuNPs, the solution was centrifuged at 12,000 rpm at room temperature for 20 min. The supernatant was removed, and the pellet was resuspended in Milli Q water (18.25 MΩ·cm). After confirmation of successful synthesis of C-AuNPs via UV-Vis, the colloidal solution was stored at 4 °C to avoid further aggregation.

### 2.2. Characterization of the C-AuNPs

The colloidal solution of 100 pM was used for the UV-Vis and Zeta potential analysis of C-AuNPs. Absorption measurements were performed with Nanodrop UV-Vis Spectrophotometer (Thermo Scientific, Loughborough, UK). A wavelength range of 300–800 nm with an increment of 0.5 nm was applied to absorb UV-Vis at room temperature. Surface charge density (Zeta potential) was measured with a zeta sizer (Malvern Zetasizer, Nano ZS-90, Malvern, UK) by using disposable cells DTS 1070. Average surface charge density was calculated by taking a mean of three measurements. TEM at 197 kV was performed on a JEOL TEM 2100 LaB_6_ thermionic gun, USA. The samples for TEM analysis were prepared by adopting the drop-casting method. ImageJ software was employed to obtain the average size by generating a histogram of C-AuNPs.

### 2.3. Optimization of Reagents and Reactions

The factors involved in the enzymatic hydrolysis of ACh and the chromogenic reaction (oxidation) of TMB by C-AuNPs were optimized. These include the pH for enzymatic and chromogenic reactions, incubation time for enzymatic hydrolysis, and the respective concentrations of ACh, TMB, and C-AuNPs. Relative absorbance ∆A (∆A = A_0_ − A) was used for the optimization, where A_0_ and A are the absorbances at 652 nm in the absence and presence of AChE respectively. Details of the optimization procedure are reported in the Supplementary Materials (Sections S1 and S2).

### 2.4. Protocol for AChE Assay via TMB Oxidation

To determine the hydrolysis of ACh by AChE, stock solution (0.5 U·mL^−1^) and working solution (2.0 m U·mL^−1^) of AChE were prepared using 20 mM Tris–HCl buffer (pH 7.5). Similarly, working solutions of ACh (4.0 mmol·L^−1^) and TMB (0.4 mmol·L^−1^) were made using Milli Q water and 100 mM sodium acetate buffer (pH 4.0), respectively. The assay was established by pipetting 50 µL of Tris-HCl buffer (100 mM, pH 7.6), 25 µL of AChE (2 mU·mL^−1^), and different concentrations of 20 µL ACh in a micro-centrifuge tube and incubated for 20 min at 37 °C. Then 50 µL of sodium acetate buffer (100 mM, pH 4.0), 25 µL of 0.4 mmol·L^−1^ TMB, 20 µL of C-AuNPs colloidal solution (100 pM), and 20 µL of the above solution were mixed thoroughly. The UV-Vis spectrums of the resultant solutions were measured, and the decrease in absorption at 652 nm depicting the aggregation effect of choline as an enzymatic product of AChE activity was measured.

### 2.5. Protocol for Sensing of Parathion Ethyl by C-AuNPs Probe

The insecticide (PE) was utilized as a candidate OP that potentially inhibits the functionality of AChE. Since OPs are neurotoxins, all the experiments were performed under the standard biosafety and chemical safety procedures with appropriate personal protective equipment (PPE). Different dilutions of PE (20, 40, 60, 80, 100, 200, 300, and 400 nM) were prepared by ethyl alcohol (95%). The 50 µL of Tris-HCl buffer (100 mM, pH 7.6), 25 µL of AChE (2 mU·mL^−1^), and different dilutions of PE (25 µL) were mixed and incubated for 10 min at 37 °C. Then 20 µL of ACh (4 mmol·L^−1^) was added to each sample, and the reagents were incubated for an additional 20 min. Then, 20 µL of sodium acetate buffer (100 mM, pH 4.0), 25 µL of 0.4 mmol·L^−1^ TMB, 25 µL of C-AuNPs (100 pM), and 20 µL of the above solution were dispensed using a micropipette. The UV-Vis spectra of the resultant solutions were recorded by an increase in the absorption at 652 nm, which was directly proportional to the concentration of PE. The inhibition efficiency (IE%) was calculated using the formula noted in the Supplementary Materials (Section S3). To work out the cross-reactivity of the designed probe, environmental contaminants such as PFOS, PFOA, GenX, cancer-causing agent imidazole, and other toxins were introduced at the concentration of 100 nM.

**Figure 1:**
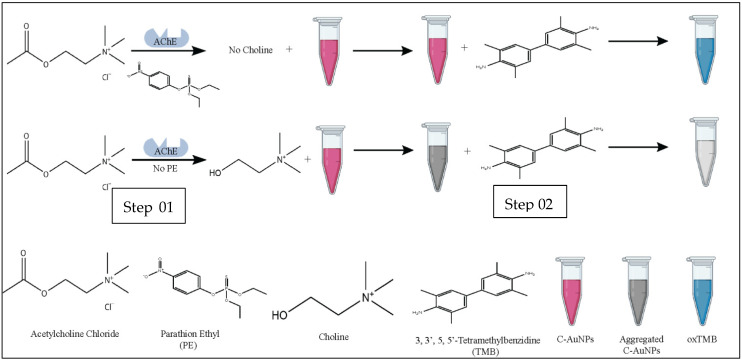
Schematic representation of colorimetric detection of PE via C-AuNPs. Step 01 represents the effect of PE on the formation of choline. Step 02 represents the oxidation potential of dispersed and aggregated C-AuNPs.

The overall methodology of the current study is summarized in Figure 1.

## 3. Results and Discussion

### 3.1. Characterization of Cysteamine Capped AuNPs

**Figure 2:**
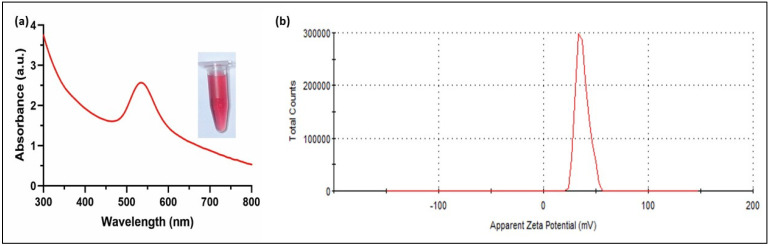
Characterization of C-AuNPs (**a**) UV-visible spectrum with maximum absorbance at 530 nm, Inset micro-centrifuge tube represents the wine red colloidal solution of freshly synthesized C-AuNPs (**b**) Zeta Potential denotes the surface charge of +39.4 ± 2.05 mV (average of three readings).

The UV-Vis absorbance spectra showed a characteristic sharp peak at 530 nm for the C-AuNPs due to the strong surface plasmon resonance (SPR) of these plasmonic NPs (Figure 2). The narrow SPR band of NPs is associated with the spherical mono-disperse materials [43]. Ganapuram et al. and Sobczak-Kupiec et al. observed similar maxima of SPR peaks for AuNPs [44,45]. The mono-dispersed nature of the C-AuNPs was further established by a high positive surface charge (+39.4 ± 2.05 mV), which contributes to the stability of these NPs because of the electrostatic repulsion of the highly positive surfaces minimizing aggregation. Similarly, the positive charge also signifies the presence of amine groups (NH_3_^+^ group) on the surface of these NPs as a capping agent [42,46].

**Figure 3:**
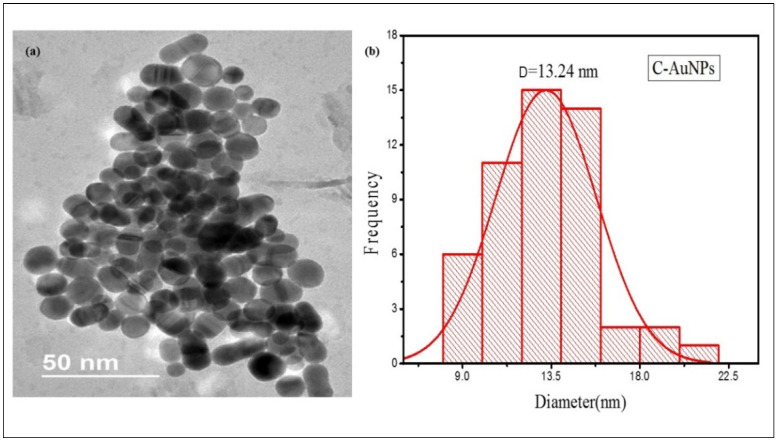
TEM micrograph of C-AuNPs (**a**) TEM images of C-AuNPs depicting spherical morphology (**b**) Size distribution histograms for C-AuNPs denoting an average size of C-AuNPs as 13.24 nm.

TEM micrograph of Figure 3a illustrates that nearly every particle is spherical with smooth surfaces. The C-AuNPs overlap with each other rendering the assembly of the ploy-dispersed NPs, representing an extensive network of small particles which is in agreement with the mono-dispersed NPs shown in TEM scans presented by Shah et al. [47]. As sample preparation for TEM analysis was done by the drop-casting method, so mono-dispersed NPs are getting interconnected, contributing to agglomeration. Evaporation of water from the sample leads to the unexpected agglomeration of the sample on the TEM grid due to surface tension during the air-drying step, though it is a temporary clustering [48,49], Since these bond formations are reversible, the shape and size of the individual NPs remain unchanged [50]. According to the histogram (Figure 3b), the average size of these NPs are only 13.24 nm which shows the application potential of this miniature size C-AuNPs, since smaller NPs have robust and better activity compared to large size AuNPs [51,52,53].

### 3.2. Optimization of Reagents and Reactions

The AChE maximally hydrolyzed ACh (4.0 mmol·L^−1^) after incubating for 20 min with Tris HCl buffer (pH 7.6) as a reaction medium sketched in the Supplementary Materials (Section S1). The 100 pM of C-AuNPs best oxidized the 0.4 mmol·L^−1^ TMB in acetate medium (pH 4.0) see Supplementary Materials (Section S2). Recently, Yan et al. and Sun et al. considered the optimization of concentrations of reagents, pH of buffer solutions, and incubation milieus as preliminary experiments for the development of a successful colorimetric assay [20,54]. The C-AuNPs have easy synthesis protocols, have more substantial catalytic power, and C-AuNPs + TMB assay needs less incubation time, making the overall process simple, easy, and quick.

### 3.3. AChE Assay via TMB Oxidation

**Figure 4:**
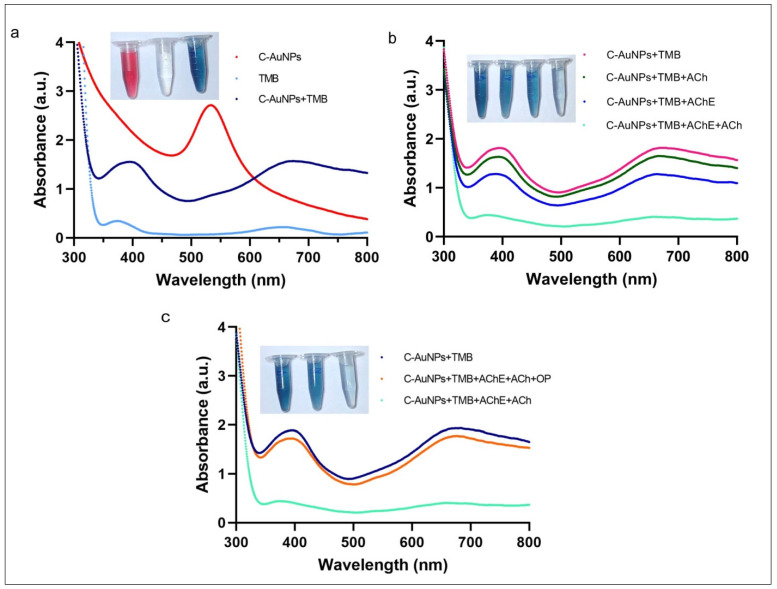
Nanozymic nature of C-AuNPs, affected by choline and PE (**a**) The UV-Vis absorption spectra of C-AuNPs, TMB, and C-AuNPs + TMB confirm the formation of oxTMB. (**b**) UV-Vis absorption spectra confirm that the appearance of choline due to AChE and ACh ultimately causes the aggregation of C-AuNPs. (**c**) The difference in UV-vis spectra in the presence and absence of PE confirms choline production, which leads to the reduction in the catalytic activity of C-AuNPs.

The peroxidase-like activity of C-AuNPs and the adverse effect of choline on the catalytic capabilities of these nanozymes were studied by utilizing TMB as a typical peroxidase substrate [55]. The red spectra in Figure 4a represent the UV-Vis spectra of C-AuNPs with a maximum peak at 530 nm. The aqua colour spectra with very shallow peaks at 370 nm and 652 nm are the UV-Vis spectra of colourless TMB where the dark blue spectra were obtained when C-AuNPs were incubated with TMB, in the presence of H_2_O_2_. The incidence of two sharp peaks confirmed the oxidation of TMB at 370 nm and 652 nm as shown in dark blue spectra. A noticeable change in the colour of TMB (colourless to intense blue) further confirms the oxidation of TMB within 60 sec at room temperature. This instant oxidation of TMB demonstrates the nanozymic nature of C-AuNPs and indicates the potential of the C-AuNPs + TMB system for the development of a quick monitoring assay. A similar observation was reported earlier by Liu et al. [56]. The inset figure of Figure 4a shows the colloidal solution of C-AuNPs (wine red), TMB (Colourless), and oxidized TMB (oxTMB) (Intense blue).

To analyse the effects of choline on the C-AuNPs + TMB system, the possible combinations of the enzyme (AChE) and substrate (ACh) were made to rule out the false-negative results and ratify the proposition that the presence of choline induces aggregation in C-AuNPs. Results obtained by these combinations suggest that the TMB is being oxidized in all combinations where both reagents, i.e., substrate and enzyme, are not present together. The prominent peaks at 370 nm and 652 nm, as shown in Figure 4b, confirm the presence of oxTMB. The oxidation of TMB advocates for the existence of well-dispersed C-AuNPs. On the other hand, when both AChE and ACh are present, the TMB oxidation was not significantly observed, which is indicated by the colourless reagent in the inset diagram of Figure 4b, and further confirmed by the spectra with no significant peaks (Figure 4b). Thus, to produce choline, both the substrate and enzyme should be present with all other required conditions and the presence of choline induced aggregation in C-AuNPs.

The aggregation of C-AuNPs due to choline has also been reported by El et al. [57]. The reason behind this aggregation depends upon the deprotonation of cysteamine under the influence of pH. Capping agents (amine groups) of C-AuNPs are being deprotonated at alkaline pH [58]. The positively charged choline acts as a bridge among deprotonated C-AuNPs and leads to the aggregation of these NPs. The aggregated form of C-AuNPs loses its catalytic activity and is unable to catalyse the oxidization of TMB. Depending upon the pH of the medium, cysteamine exists in three ionic forms: the positively charged form (cys+), the zwitterionic form (cys-ZW), and the negatively charged form (cys-) [59].

To study the effect of PE on the above-discussed C-AuNPs + TMB system, PE was introduced to block the enzymatic activity of AChE. The presence of PE in the reaction mixture reduced the Ach hydrolysis; the lesser the hydrolysis, the lower the choline production will be. The presence of lesser choline will reduce the aggregation of C-AuNPs. However, the non-aggregated C-AuNPs will remain catalytically active and exhibit peroxidase-like activity. This non-aggregated and mono-dispersed state of C-AuNPs was indicated by the blue colour of the chromogenic reaction, which was confirmed by the prominent peaks at 370 nm and 652 nm, as shown in Figure 4c.

### 3.4. Sensing of Parathion Ethyl through C-AuNPs Probe

**Figure 5:**
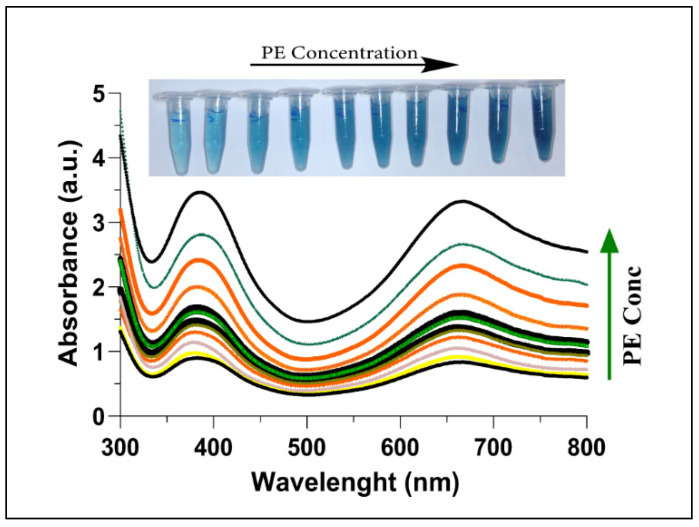
UV-Vis absorption spectra of AChE + Ach + C-AuNPs + TMB plotted with increasing concentrations of PE (0–400 nM). The increase in PE concentration leads to less aggregation of C-AuNPs, thus a stronger catalysed colorimetric reaction with deeper colour from oxTMB. The inset shows the colour of the corresponding reaction mixtures.

While characterizing the inhibition of AChE by PE, a reduced amount of choline production was observed as the presence of PE irreversibly inhibited the AChE consequently. With an increase in the concentration of PE, a gradual inactivation pattern of AChE was observed, which eventually led to higher oxidation of TMB (Figure 5). The transformation in colour (inset Figure 5) further explains the continuing oxidation of TMB, which is in agreement with Ren et al. [60]. Irreversible inhibition of AChE has already been reported due to the presence of OPs [61,62,63]. The formation of a covalent bond with the active site (phosphorylate serine residues) of AChE by these toxic chemicals converts the enzyme into a non-functioning molecule [64].

### 3.5. Effect of C-AuNPs Concentration on Oxidation of TMB

**Figure 6:**
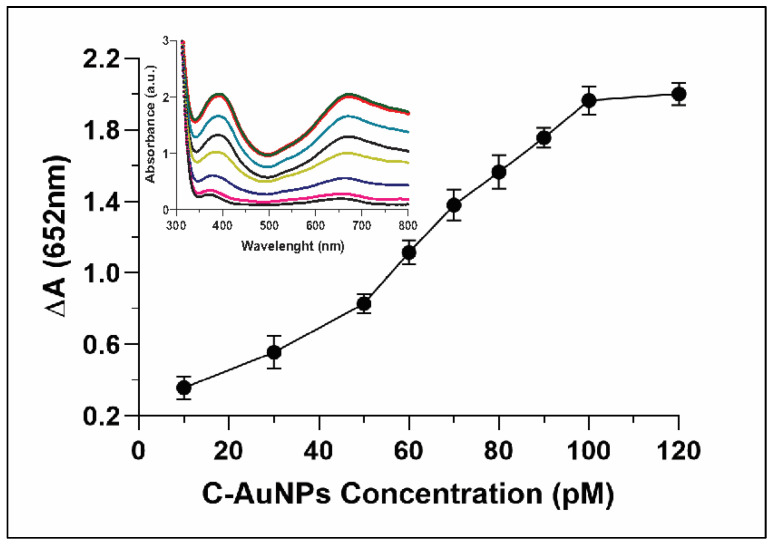
Explicate the catalytic activity of C-AuNP increasing with increasing concentrations. Inset represents the UV-Vis spectra of C-AuNPs and TMB with growing concentrations of C-AuNPs.

Whereas evaluating the correlation between concentrations of C-AuNP and oxidation of TMB, the relative absorbance ∆A (652nm) was only 0.3 when the concentration of C-AuNPs was just 10 pM. The ∆A reached up to 2.0 when the concentration of the C-AuNPs was increased to 100 pM (Figure 6). The inset in Figure 6 indicated continuous growth in the absorbance spectra with a gradual increase of the peak at 652 nm. A direct relationship was observed between the NPs concentrations and oxidation of TMB. Hence, the higher catalytic power of nanozyme was established with increasing concentration. Sun et al. also reported that in comparison to low concentration, high concentrations of nanomaterials have higher catalytic power to catalyse the oxidation of TMB [54].

### 3.6. The Efficiency of the C-AuNP Based Sensor

**Figure 7:**
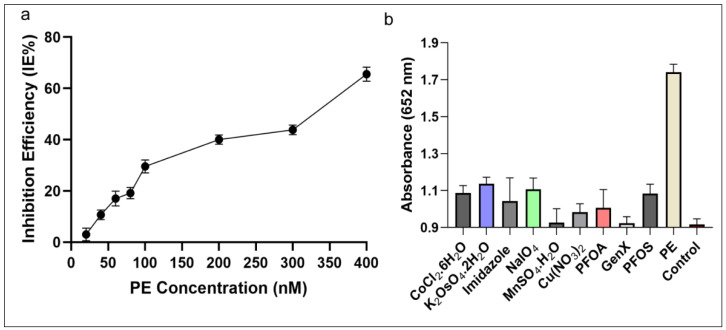
Inhibition efficiency and specificity of the assay (**a**) The IE% of PE on AChE activity demonstrates that with growing concentration of PE the inhibition of AChE increasing (**b**) The selectivity of the developed assay towards PE, carcinogenic compounds, and other toxins at a wavelength of 652 nm. The absorbance of PE was much higher compared to other compounds tested.

The IE% of AChE was detectable in the tested range between 20 and 400 nM concentration as shown in Figure 7a. The inhibitor (PE) showed a linear correlation in the range between 11.6–92.8 ng·mL^−1^ (40–320 nM), which is in agreement with the previously described studies [20,54]. The lower limit of detection of this C-AuNPs + TMB assay has been established as 5.8 ng·mL^−1^ (20 nM), which is better than several previous reports [34,35,40,41]. The LOD of classical analytical methods is comparable [36] and better [32,33] than our proposed system; however, these methods are less robust and expensive. Their electrochemical sensing approaches [37,39] have additional steps to follow long protocols and need high temperatures for hours to successfully synthesize nanomaterials. The protocol of gold nanoclusters-anchored MnO_2_ composite also needs 4–5 h to complete the synthesis in addition to other steps of the protocol [38]. Compared to nanozyme based biosensing [20] which capitalized the MnO_2_ sheets as nanozyme, the synthesis of MnO_2_ is complex and needs heating at 90 °C for an additional 15 min followed by MnO_2_ + TMB based detection, which needed more prolonged incubations. Contrarily, our protocol involves the simple synthesis of C-AuNPs, which is easy to follow, and all steps occur at room temperature. Our protocol requires 85–95 min, which includes synthesis of particles and sensing of the analytes. Thus we can say that in terms of simplicity, economics, easiness of procedure, and robustness of the protocol, the C-AuNPs + TMB sensing is better than all these reports.

The cross-reactivity assay results shown in Figure 7b demonstrate that none of the tested toxins induce the same colorimetric response as PE when presented at a concentration as high as 100 nM instead of PE (100 nM). Accordingly, these outcomes indicate that the C-AuNPs + TMB probe is specific enough to detect PE selectively with high precision and reliability. Compared to sensor technologies that utilize parathion-methyl as an analyte, our approach exhibited 180 times better sensitivity (5.8 ng·mL^−1^) compared to the MPH enzyme-based biosensor (1052.8 ng·mL^−1^) [35], and three times better than the QD-based sensor (18.0 ng·mL^−1^) [34]. The sensitivity achieved is comparable to the sensitivity of Fe_3_O_4_ imprinted polymers (5.2 ng·mL^−1^) [36], as described in Table 1. Overall, the developed assay was simple, sensitive, and highly selective in detecting OPs. Thus, our proposed sensor meets the detection requirements for on-site and robust visual detection of OPs with high sensitivity, precision, and reproducibility.

## 4. Conclusions

A simple and sensitive sensor assay based upon a nanozyme (C-AuNPs) and acetylcholinesterase inhibition by parathion ethyl (PE) was developed. The assay meets the detection requirements for on-site and robust visual monitoring of OPs with high sensitivity, precision, and reproducibility. The biosensor utilizes C-AuNPs to catalyse the oxidization of colourless TMB into a blue-coloured reagent for visual monitoring of PE with excellent specificity. The visual monitoring makes this method more adaptable than other analytical techniques which are dependent upon advanced instrumentations. This colorimetric detection system can be utilized for a range of applications to monitor PE in environmental and food processing applications such as irrigation water, juices, milk products, fruit, and vegetables for quality control, and safety purposes.

## Figures and Tables

**Table 1:** Summarizes different methods that are devised for the detection of OP compounds.

	Method	Linear Range (ng·mL^−1^)	LOD (ng·mL^−1^)	Target Analyte	References
1	LC-MS (Conventional)	--	0.5	Glyphosate	[32]
2	Enzyme-linked immunosorbent assay (Conventional)	0.44–8.48	0.19	Parathion	[33]
3	3QD based sensor (Fluorescence)	25–3000	18.0	Parathion-Methyl	[34]
4	Biosensor using MPH enzyme (Optical)	0–26, 312	1052.8	Parathion-Methyl	[35]
5	Fe_3_O_4_ imprinted polymers (Conventional)	15–2500	5.2	Parathion-Methyl	[36]
6	MIP-B-TiO_2_NRs- Voltammetry (Photoelectrochemical)	0.01–100	7.4 × 10^−3^	Chlorpyrifos	[37]
7	AuNCs-MnO_2_ based system (Fluorometric and colorimetric)	0.125–750	0.125	Carbaryl	[38]
8	AChE/AuNPs based system (Electrochemical)	0.01–1.0	2.78 × 10^−5^	Malathion	[39]
9	RB-AuNPs based system (Colorimetric and fluorescent)	969.2–3632.5	8.965	Ethoprophos	[40]
10	Aptamer-AuNPs (Colorimetric)	21.31–2130	2.1 × 10^7^	Omethoate	[41]

## Data Availability

Not applicable.

## Supplementary Materials

The following are available online at https://www.mdpi.com/article/10.3390/s21238050/s1, S1 is providing the information of optimization steps for enzymatic hydrolysis of Ach byAChE. Figure S1: Optimization of the enzymatic reaction. (a) Enzymatic hydrolysis of ACh was noted to be the best at a pH of 7.6 (b) Selection of optimum incubation time (c) Maximum ∆A at 652 nm was obtained with a 4.0 mmol·L^-1^ concentration of ACh. Error bars represent the standard deviation from three replicates; S2 contains the data about the optimization reactions conducted to get the maximum nanozymic potentials of C-AuNPs. Figure S2: Optimization of chromogenic reaction. (a) The optimum ∆A of the chromogenic reaction was at a pH of 4.0. (b) The maximum ∆A of the reaction was noted at 0.4 mmol·L^-1^. (c) The maximum ∆A was observed at 100 pM concentration of C-AuNP. Error bars represent the standard deviation of three replicates. S3: Providing the details of the applied formula for the calculation of inhibition efficiency.
