# Supplementary material for: Colorimetric Detection of Organophosphate Pesticides Based on Acetylcholinesterase and Cysteamine Capped Gold Nanoparticles as Nanozyme
Source: Sensors (Basel). 2021 Dec 2;21(23):8050. doi: 10.3390/s21238050 (PMC8659924; doi:10.3390/s21238050)
Supplement: Supplementary file 1 [file sensors-21-08050-s001.zip › sensors-1438555-supplementary.pdf]

sensors

MDPI

Article

Colorimetric Detection of Organophosphate Pesticides Based on Acetylcholinesterase and Cysteamine Capped Gold Nanoparticles as Nanozyme

Muhammad Musaddiq Shah, Wen Ren, Joseph Irudayaraj, Abdulrahim A. Sajini, Muhammad Ishtiaq Ali and Bashir Ahmad

S1. Optimization of Enzymatic Reaction

Experimental conditions which can influence the enzymatic product of AChE were optimized one by one. Tris-HCl buffer was capitalized as a reaction medium for the enzymatic hydrolysis of ACh. Factors including the pH of the buffer, incubation time, and the concentration of ACh were considered for optimization.

S1 (a). Optimization of pH for Tris-HCl Buffer

Experimental constants for optimization of the buffer

i. Enzymatic hydrolysis: (AChE 2.0 mU·mL^{-1}, ACh 4.0 mmol·L^{-1}, Incubation temp 37°C, Incubation time 20 min)

ii. Chromogenic reaction: (Sodium acetate buffer pH 4.0, TMB concentration 0.4 mmol·L^{-1}, C-AuNPs concentration 100 pM)

The pH was optimized in the range 7.0-8.4 using Tris-HCl buffer. The ΔA at 652 increased with an increase in pH initially, reaching maxima at a pH of 7.6. Further increase in pH leads to a decrease in the activity of the enzyme, as depicted in Figure S1 (a), hence a pH of 7.6 was considered as the optimum pH for upcoming biosensing assay.

S1 (b). Optimization of Incubation Time

Experimental constants for optimization of incubation time

i. Enzymatic hydrolysis: (AChE 2.0 mU·mL^{-1}, ACh 4.0 mmol·L^{-1}, Incubation temp 37°C, Tris-HCl buffer pH 7.6)

ii. Chromogenic reaction: (Sodium acetate buffer pH 4.0, TMB concentration 0.4 mmol·L^{-1}, C-AuNPs concentration 100 pM)

Another critical factor besides the pH of the reaction was the incubation time. The reaction mixture was incubated for 10-60 min at 37°C with 10-min intervals (Figure S1(b)). The ΔA at 652 nm indicates that at 20 min of incubation, maximum product was obtained. Any further extension of the incubation time did not affect the concentration of choline considerably since the peak stabilized. Hereafter 20 min was selected as the incubation time for maximum choline production.

S1 (c). Optimization of ACh Concentration

Experimental constants for optimization of ACh concentration

i. Enzymatic hydrolysis: (AChE 2.0 mU·mL-1, Incubation temp 37°C, Tris-HCl buffer pH 7.6, Incubation time 20 min)

ii. Chromogenic reaction: (Sodium acetate buffer pH 4.0, TMB concentration 0.4 mmol·L-1, C-AuNPs concentration 100pM)

Minimum substrate concentration with the maximum product of choline was examined by selecting the range of concentrations from 2 to10 mmol·L-1 of the substrate with an interval of 2 mmol·L-1 (Figure S1(c)). A concentration of 4.0 mmol·L-1 of ACh was found to be the optimal concentration and used as a standard concentration throughout the assay.

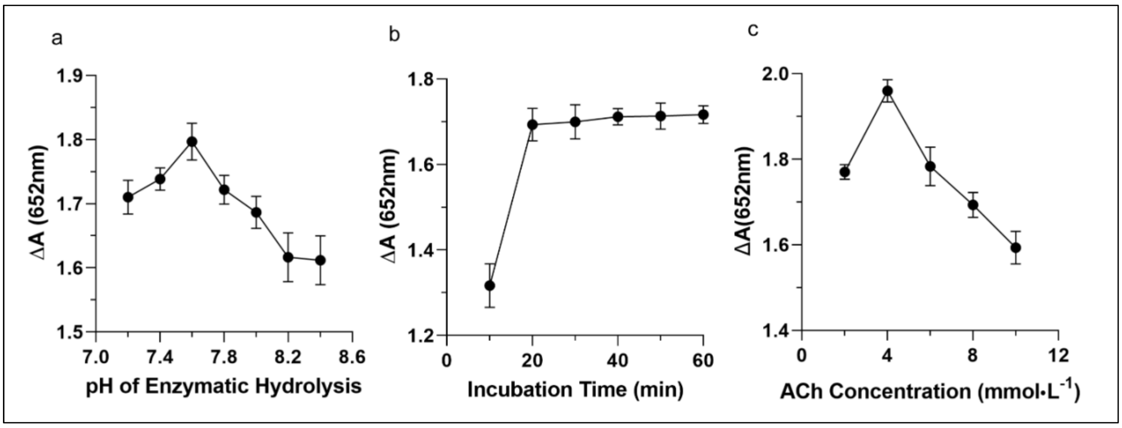

Figure S1 Optimization of the enzymatic reaction. (a) Enzymatic hydrolysis of ACh was noted to be the best at a pH of 7.6 (b) Selection of optimum incubation time (c) Maximum ΔA at 652 nm was obtained with a 4.0 mmol·L-1 concentration of ACh. Error bars represent the standard deviation from three replicates.

S2. Optimization of the Chromogenic Reaction

The chromogenic reaction was conducted to assess the enzymatic potential of C-AuNPs. Sodium acetate buffer was consumed as the reaction medium for this reaction. To determine the optimum condition for the reaction, different preliminary experiments were performed to determine the optimum pH, the best concentration of TMB, and the concentration of C-AuNPs.

S2 (a). Optimization of pH for Sodium Acetate Buffer

Experimental constants for optimization of the buffer pH

i. Enzymatic hydrolysis: (AChE 2.0 mU·mL-1, ACh 4.0 mmol·L-1, Incubation temp 37°C, Tris-HCl buffer pH 7.6, Incubation time 20 min)

ii. Chromogenic reaction: (TMB concentration 0.4 mmol·L-1, C-AuNPs concentration 100pM)

A pH of 3.0 to 5.0 was selected to determine the best pH for this reaction. The absorbance variation (ΔA) was monitored at 652 nm against different pH. It was noted that the reaction produced the maximum ΔA at 652 nm when the pH of the buffer was 4.0 (Figure S2(a)). Hence a pH of 4.0 was utilized as a standard pH for all chromogenic reaction-based experiments.

S2 (b). Optimization of TMB Concentration

Experimental constants for optimization of TMB concentration

i. Enzymatic hydrolysis: (AChE 2.0 mU·mL^{-1}, ACh 4.0 mmol·L^{-1}, Incubation temp 37°C, Tris-HCl buffer pH 7.6, Incubation time 20 min)

ii. Chromogenic reaction: (Sodium acetate buffer pH 4.0, C-AuNPs concentration 100pM)

The concentration range used for the optimization of TMB was from 0.2-1.0 (mmol·L^{-1}). It was noted that the concentration of 0.4 mmol·L^{-1} provided optimal results. Later, for all experiments with chromogenic reactions, 0.4 mmol·L^{-1} of TMB was added (Figure S2(b)).

S2 (c). Optimization of C-AuNPs Concentration

Experimental constants for optimization of C-AuNPs concentration

i. Enzymatic hydrolysis: (AChE 2.0 mU·mL^{-1}, ACh 4.0 mmol·L^{-1}, Incubation temp 37 °C, Tris-HCl buffer pH 7.6, incubation time 20 min)

ii. Chromogenic reaction: (Sodium Acetate buffer pH 4.0, TMB concentration 0.4 mmol·L^{-1})

To determine the effect of C-AuNPs concentrations on the oxidation of TMB, ranges of the concentrations from 10-120 pM were applied (Figure S2(c)). It was noted that the concentration of the nanoparticles was directly linked with the oxidation potential of the nanoparticle probes. Maximum absorbance was obtained at 100 pM of C-AuNPs.

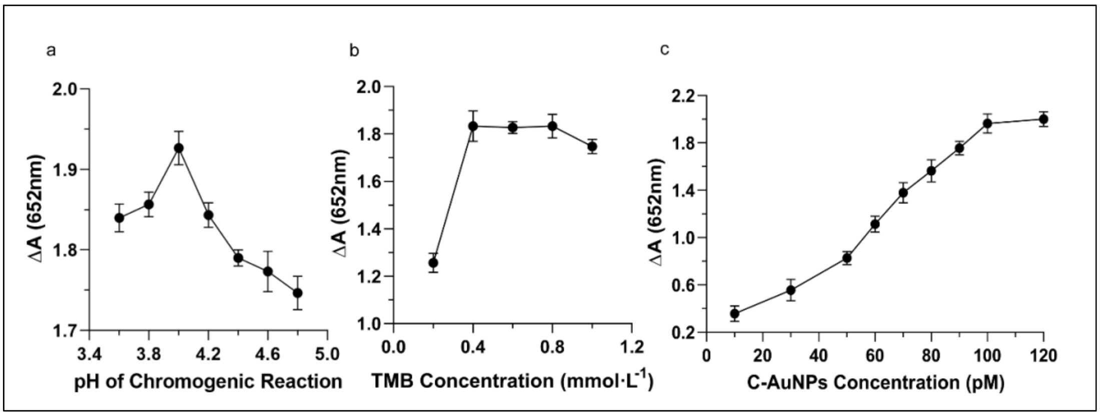

Figure S2 Optimization of chromogenic reaction. (a) The optimum ΔA of the chromogenic reaction was at a pH of 4.0. (b) The maximum ΔA of the reaction was noted at 0.4 mmol·L^{-1}. (c) The maximum ΔA was observed at 100 pM concentration of C-AuNP. Error bars represent the standard deviation of three replicates.

S3. Inhibition Efficiency

The inhibition efficiency IE (%) was used as an indicator to calculate the inhibitory effect of Parathion ethyl (PE) on AChE activity. IE (%) was calculated by applying following formula:

IE (%) = (F (inhibitor) – F (no Inhibitor)) / (F0- F (no inhibitor)) × 100%

Where

F denotes absorbance intensity at 652 nm.

F (no inhibitor) = (AChE+ACh+ C-AuNPs +TMB)

F (inhibitor)= (AChE+PE+ACh+C-AuNPs +TMB)

F0 = (C-AuNPs+TMB)

Where F (no inhibitor), F (inhibitor), and F0 are the absorbance intensities at 652 nm in the UV-Vis spectra for the above-mentioned combinations. The IE (%) was calculated for different concentrations, and it was noted that with an increase in the concentration of the PE, a gradual decrease in the activity of AChE was observed.
